# Supplementary material for: Recommendations on the use and reporting of race, ethnicity, and ancestry in genetic research: Experiences from the NHLBI TOPMed program
Source: Cell Genom. 2022 Jul 26;2(8):100155. doi: 10.1016/j.xgen.2022.100155 (PMC9481067; doi:10.1016/j.xgen.2022.100155)
Supplement: Document S1. Additional background and references [file mmc1.pdf]

Cell Genomics, Volume *2*

Supplemental information

Recommendations on the use and reporting of race, ethnicity, and ancestry in genetic research: Experiences from the NHLBI TOPMed program

Alyna T. Khan, Stephanie M. Gogarten, Caitlin P. McHugh, Adrienne M. Stilp, Tamar Sofer, Michael L. Bowers, Quenna Wong, L. Adrienne Cupples, Bertha Hidalgo, Andrew D. Johnson, Merry-Lynn N. McDonald, Stephen T. McGarvey, Matthew R.G. Taylor, Stephanie M. Fullerton, Matthew P. Conomos, and Sarah C. Nelson

Additional background

From 2014-2020, the TOPMed Data Coordinating Center (DCC) was housed in the Genetic Analysis Center (GAC) in the Department of Biostatistics at the University of Washington[1]. The GAC has performed scientific, analytical, and/or administrative coordination for a range of human genomics consortia and programs over the past 15 years including the NHGRI Gene Environment Association Studies consortium (GENEVA, 2007-2011), NHGRI Genomics and Randomized Trials Network (GARNET, 2009-2012), and the NHLBI Hispanic Community Health Study/Study of Latinos (HCHS/SOL, 2013-2016). Through these efforts, we have established standards for genotypic data quality assurance that account for population structure[2], grappled with how to analyze and report genetic diversity, e.g. among Hispanic/Latino groups[3], and developed statistical methods and software for analyzing diverse datasets[4–7]. In 2018, GAC staff initiated monthly internal discussions on the use of race, ethnicity, and ancestry in genetics research—engaging with academic literature, public media, and our own experiences working in TOPMed and prior genetics consortia. We discussed a variety of articles across disciplines[8–11] and invited guest speakers on topics such as statistical rationale for stratified analyses and the co-opting of population genetic research by white supremacists on social media[12]. Discussions were informed by a range of training and experience at the DCC, including biostatistics; statistical genetics; science communication; public health genetics; and ethical, legal, and social implications (ELSI). From these discussions, we recognized the opportunity as a DCC to help establish recommendations for TOPMed researchers that address the challenges of working with diverse data and incorporate anti-racist principles[13] into the research process.

References

1. Genetic Analysis Center https://www.biostat.washington.edu/research/centers/gac.
2. Laurie, C.C., Doheny, K.F., Mirel, D.B., Pugh, E.W., Bierut, L.J., Bhangale, T., Boehm, F., Caporaso, N.E., Cornelis, M.C., Edenberg, H.J., et al. (2010). Quality control and quality assurance in genotypic data for genome-wide association studies. Genet. Epidemiol. *34*, 591–602.
3. Conomos, M.P., Laurie, C.A., Stilp, A.M., Gogarten, S.M., McHugh, C.P., Nelson, S.C., Sofer, T., Fernández-Rhodes, L., Justice, A.E., Graff, M., et al. (2016). Genetic Diversity and Association Studies in US Hispanic/Latino Populations: Applications in the Hispanic Community Health Study/Study of Latinos. Am. J. Hum. Genet. *98*, 165–184.
4. Conomos, M.P., Miller, M.B., and Thornton, T.A. (2015). Robust inference of population structure for ancestry prediction and correction of stratification in the presence of relatedness. Genet. Epidemiol. *39*, 276–293.
5. Conomos, M.P., Reiner, A.P., Weir, B.S., and Thornton, T.A. (2016). Model-free Estimation of Recent Genetic Relatedness. Am. J. Hum. Genet. *98*, 127–148.
6. Gogarten, S.M., Sofer, T., Chen, H., Yu, C., Brody, J.A., Thornton, T.A., Rice, K.M., and Conomos, M.P. (2019). Genetic association testing using the GENESIS R/Bioconductor package. Bioinforma. Oxf. Engl. *35*, 5346–5348.
7. Chen, H., Wang, C., Conomos, M.P., Stilp, A.M., Li, Z., Sofer, T., Szpiro, A.A., Chen, W., Brehm, J.M., Celedón, J.C., et al. (2016). Control for Population Structure and Relatedness

for Binary Traits in Genetic Association Studies via Logistic Mixed Models. Am. J. Hum. Genet. *98*, 653–666.
8. Reich, D. (2018). How Genetics Is Changing Our Understanding of ‘Race.’ N. Y. Times.
9. Roth, W.D., and Ivemark, B. (2018). Genetic Options: The Impact of Genetic Ancestry Testing on Consumers’ Racial and Ethnic Identities. Am. J. Sociol. *124*, 150–184.
10. Royal, C.D., Novembre, J., Fullerton, S.M., Goldstein, D.B., Long, J.C., Bamshad, M.J., and Clark, A.G. (2010). Inferring Genetic Ancestry: Opportunities, Challenges, and Implications. Am. J. Hum. Genet. *86*, 661–673.
11. Mills, M.C., and Rahal, C. (2019). A scientometric review of genome-wide association studies. Commun. Biol. *2*, 9.
12. Carlson, J., and Harris, K. (2020). Quantifying and contextualizing the impact of bioRxiv preprints through automated social media audience segmentation. PLOS Biol. *18*, e3000860.
13. Brothers, K.B., Bennett, R.L., and Cho, M.K. (2021). Taking an antiracist posture in scientific publications in human genetics and genomics. Genet. Med.
