# Supplementary material for: Recommendations on the use and reporting of race, ethnicity, and ancestry in genetic research: Experiences from the NHLBI TOPMed program
Source: Cell Genom. 2022 Jul 26;2(8):100155. doi: 10.1016/j.xgen.2022.100155 (PMC9481067; doi:10.1016/j.xgen.2022.100155)
Supplement: Document S2. Article plus supplemental information [file mmc2.pdf]

Cell Genomics

CellPress
OPEN ACCESS

Perspective

Recommendations on the use and reporting of race, ethnicity, and ancestry in genetic research: Experiences from the NHLBI TOPMed program

Alyna T. Khan,[1,2,16,17,*] Stephanie M. Gogarten,[1,16] Caitlin P. McHugh,[1] Adrienne M. Stilp,[1] Tamar Sofer,[3,4] Michael L. Bowers,[1] Quenna Wong,[1] L. Adrienne Cupples,[5,6] Bertha Hidalgo,[7] Andrew D. Johnson,[8,9] Merry-Lynn N. McDonald,[10,11] Stephen T. McGarvey,[12,13] Matthew R.G. Taylor,[14] Stephanie M. Fullerton,[15] Matthew P. Conomos,[1] and Sarah C. Nelson[1,2,*]

[1]Department of Biostatistics, University of Washington, Seattle, WA, USA
[2]Institute for Public Health Genetics, University of Washington, Seattle, WA, USA
[3]Department of Medicine, Harvard Medical School, Boston, MA, USA
[4]Division of Sleep and Circadian Disorders, Brigham and Women's Hospital, Boston, MA, USA
[5]Department of Biostatistics, Boston University School of Public Health, Boston, MA, USA
[6]Department of Epidemiology, Boston University School of Public Health, Boston, MA, USA
[7]Department of Epidemiology, University of Alabama at Birmingham, Birmingham, AL, USA
[8]Population Sciences Branch, Division of Intramural Research, National Heart, Lung and Blood Institute, Framingham, MA, USA
[9]The Framingham Heart Study, Framingham, MA, USA
[10]Department of Medicine, University of Alabama at Birmingham, Birmingham, AL, USA
[11]Department of Genetics, University of Alabama at Birmingham, Birmingham, AL, USA
[12]Department of Epidemiology and International Health Institute, Brown University School of Public Health, Providence, RI, USA
[13]Department of Anthropology, Brown University, Providence, RI, USA
[14]Department of Medicine, Adult Medical Genetics Program, University of Colorado Anschutz Medical Campus, Aurora, CO, USA
[15]Department of Bioethics & Humanities, University of Washington, Seattle, WA, USA
[16]These authors contributed equally
[17]Lead contact
*Correspondence: alynak@uw.edu (A.T.K.), sarahcn@uw.edu (S.C.N.)
https://doi.org/10.1016/j.xgen.2022.100155

SUMMARY

How race, ethnicity, and ancestry are used in genomic research has wide-ranging implications for how research is translated into clinical care and incorporated into public understanding. Correlation between race and genetic ancestry contributes to unresolved complexity for the scientific community, as illustrated by heterogeneous definitions and applications of these variables. Here, we offer commentary and recommendations on the use of race, ethnicity, and ancestry across the arc of genetic research, including data harmonization, analysis, and reporting. While informed by our experiences as researchers affiliated with the NHLBI Trans-Omics for Precision Medicine (TOPMed) program, these recommendations are applicable to basic and translational genomic research in diverse populations with genome-wide data. Moving forward, considerable collaborative effort will be required to ensure that race, ethnicity, and ancestry are described and used appropriately to generate scientific knowledge that yields broad and equitable benefit.

INTRODUCTION

Heeding the well-founded calls to increase diversity in genomic research[1,2] requires researchers to appropriately conceptualize, use, and report on race, ethnicity, and ancestry. Indeed, the role of race in genomic and other biomedical research is a widely discussed and historically fraught issue.[3–8] The National, Heart, Lung, and Blood Institute (NHLBI) Trans-Omics for Precision Medicine (TOPMed) program provides a compelling and concrete use case to grapple with such issues, comprising over 80 contributing studies with diversity in terms of populations, geographic locations, genetic ancestries, and areas of phenotypic focus.[9] Below, we elaborate on the challenges and opportunities for the genomics research community in analyzing diverse and heterogeneous datasets and our approach in the TOPMed program.

While the field of human genetics may have reached consensus that race is a socio-political rather than biological construct,[10] the correlation between race and genetic ancestry—in that racial categories are often enriched for specific ancestries[11]—continues to complicate scientific and public discourse. Studies show that genomics professionals use heterogeneous definitions and applications of race and ancestry in research and practice[12–15] and that such scientific uses evolve in broader social and political contexts.[16] In addition, the tendency to categorize ancestry at the continental level leads to conflation with the concept of biological

This is an open access article under the CC BY license (http://creativecommons.org/licenses/by/4.0/).

race.[17] Race and ethnicity are still misused to avoid confounding due to genetic ancestry,[16,18] despite alternate approaches.[19,20] Overall, lack of agreement in the genomics research community has led to an *ad hoc* collection of research practices, with negative implications including reification of race as a biological construct[21–23] and over-attribution of health disparities to genetic rather than social and structural causes.[24–27]

To address the challenges noted above, investigators affiliated with the TOPMed program created a set of recommendations on the use of race, ethnicity, and ancestry when analyzing genome-wide data. These recommendations are organized by chronology of a standard research process: terminology (assessing what data is available for analysis and the population nomenclature), harmonization (combining and standardizing race, ethnicity, and ancestry variables across datasets), analysis (conducting and interpreting association analyses), and reporting (communicating the findings). We do not address prospective data collection, as TOPMed utilizes pre-existing phenotype data. We discuss below the common applications of race, ethnicity, and ancestry in each stage of research, the challenges we observed, and recommendations for how to move forward.

BACKGROUND

We are researchers affiliated with the NHLBI TOPMed program motivated to conduct scientifically robust and ethically responsible genetic research that leads to equitable benefit. Our prior experiences working with human genomics consortia and discussion of relevant literature and media (see details in supplemental information) led us to establish recommendations for TOPMed researchers that address the challenges of working with diverse data and incorporate anti-racist principles[8] into the research process. Here, we present recommendations developed for the use and reporting of race, ethnicity, and ancestry in TOPMed, which are broadly applicable to genetic research in diverse populations (described below and summarized in Box 1).

TOPMed as a motivating use case

TOPMed is a large consortium of ongoing "omic" (i.e., genomic, transcriptomic, proteomic, metabolomic, and methylomic) studies that encompass people of different races, ethnicities, geographic locations, and ancestries.[9] TOPMed comprises >80 studies based within and outside of the US, including founder populations such as Samoan and Amish. Broadly, TOPMed participants are 41% European ancestry (European, European American), 31% African ancestry (African, African American, African Caribbean), 15% Hispanic/Latino (including Mexican, Mexican American, Central American, South American, Cuban, Dominican, Puerto Rican), 9% Asian ancestry (Chinese, Taiwanese, Asian American, Pakistani), and 4% "other" (Samoan, Native American, multiple, or unknown).[9] This diversity enables the expansion of knowledge of genetic variation and an improved understanding of disease.[28] For example, 78.7% of 400 million variants observed in TOPMed were not previously deposited in dbSNP.[9]

Establishing recommendations for TOPMed

We created recommendations for TOPMed investigators to encourage researchers to make well-founded and responsible analytical and methodological decisions when using race, ethnicity, and ancestry variables and to communicate these concepts in an informed, transparent, and respectful manner. These recommendations were discussed in relevant TOPMed Committees (Ethical, Legal, and Social Issues [ELSI] and Analysis), approved by the TOPMed Executive Committee, and presented at consortium-wide meetings. However, they do not represent official TOPMed policy or a consensus view of the over 1,000 TOPMed investigators. We solicited examples from study investigators of study-specific considerations and preferences, e.g., for population labels, and incorporated diverse expertise and experiences to make the recommendations practical, robust, and compelling for a wide audience of genetics researchers. Ultimately, these recommendations guide investigators through challenges of using socially and genetically defined groups in scientific discussions by presenting an overview of commonly used terminology, highlighting considerations for data harmonization and analysis, and providing guidance on how to report results. While developed in the context of the TOPMed program, we contend that these recommendations are relevant for genetic and biomedical researchers working in other contexts, especially those involving diverse populations and/or the genetic study of conditions that suggest health disparities.

RECOMMENDATIONS

Terminology

When presenting information on the race, ethnicity, or ancestry of participants in a study, it is essential to be clear about whether the labels used refer to reported or genetically inferred information. "Race" and "ethnicity" generally refer to social, not biological, categories, and they are often used interchangeably. In contrast, "ancestry" is generally used in genetic research to refer to one's biological ancestors from whom their DNA was inherited or to imply something about a person's genetic origins; for example, the continental origin of the majority of their ancestors (sometimes referred to as "continental ancestry").[29,30] Ancestry can also refer to having ancestors from specific countries or geographic regions and is often how ancestry is used colloquially. Here, we use the terms race and ethnicity to refer to non-biological social categories, and we use the term genetic ancestry to describe genetic origins. Because reported race or ethnicity and genetic ancestry may all be used analytically and appear in scientific discussions and communications, care must be taken to describe exactly what is being presented and why.

Recommendations for investigators include the following:

1. **Explicitly distinguish between variables that derive from non-genetic, reported information versus genetically inferred information.**
2. **Avoid using terms that are historically linked to hierarchical, racial typologies.** For example, "Caucasian" should not be used;[31,32] instead, use "White" when referring to race and "European ancestry" when referring to genetic ancestry.
3. **Follow standards from publishers, including the APA's guidelines on bias-free language regarding racial and ethnic identity[33] and the AMA Manual of Style.[34]**

Box 1. Summary of recommendations on the use and reporting of race, ethnicity, and ancestry in genomics research

1. **Terminology**
   - 1.1 Explicitly distinguish between variables that derive from non-genetic, reported information versus genetically inferred information.
   - 1.2 Avoid using terms that are historically linked to hierarchical, racial typologies.
   - 1.3 Follow standards from publishers, including the APA's guidelines on bias-free language regarding racial and ethnic identity and the AMA Manual of Style.
2. **Harmonization of race and ethnicity across studies**
   - 2.1 Clearly describe the source data for race and ethnicity information from each study when using harmonized variables.
   - 2.2 Avoid assuming that non-genetic, reported variables are by self-report.
   - 2.3 Avoid applying US race categories to participants of studies based outside of the US.
   - 2.4 Preserve specific population information when possible rather than prematurely collapsing different populations into broader categories.
3. **Analysis**
   - 3.1 Articulate and justify why race, ethnicity, or ancestry variables were used in a given analysis.
   - 3.2 Consider that while using race or ethnicity as a covariate may explain trait variation due to social factors, it may also reinforce harmful stereotypes.
   - 3.3 Avoid using reported race or ethnicity as a proxy for genetic ancestry or using genetic ancestry to represent race or ethnicity.
   - 3.4 Focus attention on pooled- or meta-analysis results of all participants.
   - 3.5 Consider potential benefits versus potential harms when thinking about whether and how to conduct a population-specific analysis.
4. **Reporting**
   - 4.1 Acknowledge the broader social context of health and healthcare disparities when invoking these disparities as a justification for genomic research.
   - 4.2 Avoid reinforcing the idea that race and ethnicity are genetic concepts when presenting genetically derived data.
   - 4.3 Describe participants in alignment with their communities' preferences and study-specific reporting guidelines.
   - 4.4 Avoid generalizing from a single population to represent another, broader population.

Harmonization of race and ethnicity across studies

Race and/or ethnicity are commonly collected by having study participants fill out a form, which leads to "self-reported" values. Other collection methods include designation by a third party (healthcare provider or study data collector) who typically infers the participant's ascriptive race or through study documents that describe the recruitment population but do not ask whether the self-reported race and/or ethnicity of specific individuals differs from the target population. Race and/or ethnicity may also be collected multiple times, for example, in a longitudinal study, which can lead to multiple values for the same participant if their self-identification changes over time. However collected, the race and/or ethnicity of a participant is almost always a function of the specific options provided in study instruments, which will often vary by location or the research interests of investigators.

The diversity in data-collection methods presents a challenge for investigators attempting to combine data from multiple studies. Unlike quantitative phenotypes that can be transformed to a single scale during data harmonization, there is often no straightforward method to convert one set of race or ethnicity categories into another. This is particularly the case when study cohorts include individuals sampled from distinct national contexts where socio-cultural understandings of racial and/or ethnic identity differ, when working with studies over different recruitment periods, or when different studies provide different options for race and ethnicity categories (such as offering the descriptor Asian on a form versus offering more specific identifiers, like "East Asian" or "South Asian"). Thus, it is important to keep in mind the complexities and nuances of social identity when attempting to harmonize race and ethnicity variables across studies.

Recommendations for investigators include the following:

1. **Clearly describe the source data for race and ethnicity information from each study when using harmonized variables.** Include details such as whether source information is self-reported or ascribed and whether multiple categories are collapsed. Be aware that cross-study harmonized variables may represent a simplification of more complex sources of information that may not translate between different studies and jurisdictions.
2. **Avoid assuming that non-genetic, reported variables are by self-report.** Study- or cohort-specific documentation may help determine whether variables (e.g., race or ethnicity) were self-reported versus recorded by study personnel without soliciting self-report from the participant.
3. **Avoid applying US race categories to participants of studies based outside of the US.** Concepts of racial and/or ethnic identity differ across countries, and approaches to capturing this information vary across geographic location and over time.[35] For example, the racial category "Black" is used by many countries but with different meanings in each country (e.g., the US and

Brazil[36,37]), so combining those categories is inappropriate. Some countries do not collect race information at all; for example, Australia abandoned the use of racial classification in 1974 and instead collects information on ethnicity.[35]

4. **Preserve specific population information when possible rather than prematurely collapsing different populations into broader categories.** We encourage retaining as granular of information as is practical during harmonization to allow flexible tailoring of downstream analysis and accurate reporting. For example, preserve detailed population descriptors such as "Chinese American" and "Pakistani" rather than harmonizing into a single Asian group.

Analysis

When considering how to use race, ethnicity, and/or genetic ancestry information in an analysis, analysts should first assess the goals of the study and the intended purpose of including those variables in models. In a genome-wide association study (GWAS), the goal is to identify genetic variants that are associated with a particular trait or disease. Race and ethnicity are often tied to social and environmental factors influencing health[38–42] and, in such cases, may explain variation in the trait or disease of interest that is dependent on aspects of social identity (e.g., that may result from systemic or individual racial discrimination) rather than genetic ancestry. For example, African Americans with a high proportion of European ancestry may suffer the same lack of access to adequate health care as African Americans with little to no European ancestry. While race and ethnicity can be, and often are, included as covariates in association models to proxy such effects,[16] this approach may inadvertently reinforce harmful stereotypes. Therefore, it is preferable to include relevant environmental or socioeconomic variables (e.g., measures of healthcare, diet, or neighborhood disadvantage) directly in association models as covariates when available. However, adjustment for covariates that explain variation independent of genotype may either increase or decrease precision of genotype effect estimates and in turn affect statistical power to detect association.[43,44] Whether and how to integrate social factors into GWASs is an evolving and unresolved discussion in the genomics community.

On the other hand, adjusting for genetic ancestry is widely accepted practice in GWASs because it reduces false positives when populations have different trait values or disease prevalences as well as different allele frequencies and patterns of linkage disequilibrium, i.e., when there is confounding due to population stratification.[20,45–47] One approach to adjust for this confounding is to perform a pooled analysis (i.e., an analysis including all study samples) and include genetic ancestry measures derived from sample genotype data as covariates. A distinct benefit of this approach is that it does not require arbitrarily clustering participants into groups or cross-study harmonization of demographic variables. Further, this approach allows for inclusion of all participants in the analysis, including those with either missing or underrepresented race or ethnicity.[48]

A popular method to measure genetic ancestry is principal-component analysis (PCA), which generates eigenvectors that represent the genetic ancestry variation among participants as a continuous, multidimensional distribution,[20] in which those with ancestors from the same geographical area often cluster together.[49] Alternatively, admixture analysis estimates the proportion of each participant's genome descended from pre-specified reference populations of known ancestry.[19] Adjusting for either of these measures in a pooled analysis can effectively control for confounding due to genetic ancestry. The continuous nature of these measures illustrates the heterogeneity in genetic ancestry among individuals who may identify as the same race or ethnicity, particularly admixed individuals. For example, those who identify as Hispanic/Latino in the US represent a wide variety of genetic ancestries, with different proportions of ancestry admixture from Africa, the Americas, Asia, and Europe.[50–52] This highlights that simply using race and/or ethnicity as a proxy for genetic ancestry, or vice versa, is problematic in that it falsely equates the two correlated, albeit distinct, concepts.

Association tests are often conducted via meta-analysis, where different racial, ethnic, or ancestry groups are stratified and analyzed separately, and summary statistics from each group are subsequently combined. The motivations for performing meta-analysis may be logistical, e.g., the inability to combine participant data due to technical or data-sharing constraints, and/or analytical, such as the desire to adjust for genetic ancestry, environmental, or socioeconomic factors separately by group. Indeed, meta-analysis can be a useful tool, but it requires careful consideration of how groups are constructed and interpreted—an issue avoided in pooled analysis. We encourage investigators who take this approach to focus on the final meta-analysis results and exercise caution when interpreting the group-specific results.

A commonly referenced motivation for stratifying and interpreting group-specific results is to determine whether participants of a particular group are "driving" the observed association signal. While a statistically significant association may be observed in one group and not another, in our experience, we contend that this is likely due to differences in statistical power to detect an association (e.g., due to sample size or allele frequency differences) rather than fundamental differences in the underlying biological impact of the same variant in different groups of people. For example, when analyzing TOPMed data, we typically have not found additional signals from group-specific analyses that were not also identified by pooled analysis including the same individuals. On the other hand, population-specific results of previously understudied populations may provide actionable findings. Therefore, it is critical to engage with study participants or representatives on whether it is appropriate to pursue population-specific analysis and how best to represent them in the study. Researchers must earn the trust of the communities involved in their research, especially in the case of minority groups who have been historically exploited in biomedical research studies and the scientific community.[53,54] Ultimately, it is important to recognize the various technical and contextual factors that influence analytical decisions and to be transparent about which approach was taken and why.

Recommendations for investigators include the following:

1. **Articulate and justify why race, ethnicity, or ancestry variables were used in a given analysis.** Explain the reasoning behind analytical decisions to use non-genetic and/or genetically inferred variables in the methods section. Analytical decisions are nuanced and often reflect a weighing of various pros and cons to different approaches.
2. **Consider that while using race or ethnicity as a covariate may explain trait variation due to social factors, it may also reinforce harmful stereotypes.** Race or ethnicity may correlate with non-genetic, social factors, but the effects of such factors can be better accounted for when used directly, if the data are available. Whether or not including such variables is statistically beneficial is nuanced and requires careful consideration.
3. **Avoid using reported race or ethnicity as a proxy for genetic ancestry or using genetic ancestry to represent race or ethnicity.** Race and ethnicity can be correlated with genetic ancestry, but they are not the same. Individuals who identify as the same race or ethnicity can have a wide variety of genetic ancestries, and individuals with similar genetic ancestry may identify as different races or ethnicities.
4. **Focus attention on pooled- or meta-analysis results of all participants.** Whether a pooled- or a meta-analysis was used may depend on logistical and/or analytical reasons. Describe which approach was taken, why, and what the limitations may be.
5. **Consider potential benefits versus potential harms when thinking about whether and how to conduct a population-specific analysis.** Consult with study representatives or documentation to understand if their study participants would find it acceptable, or even preferred, to acknowledge their unique population history and evolution. For some understudied populations, population-specific results may provide actionable findings for that population.[55,56] However, in some instances, participants may not wish to associate membership in their population with a specific trait that could be considered stigmatizing.[57]

Reporting

Reporting on race, ethnicity, and ancestry is typically necessary to describe methods, justify approach, and interpret results. Reviews of human genetic studies identified inadequate descriptions of race, ethnicity, and ancestry variables, which hinders transparency, replicability, and interpretability.[58,59] We offer guidance on the reporting of race, ethnicity, and ancestry variables to augment existing and emerging reporting recommendations (e.g., Brothers et al.,[8] American Psychological Association,[33] and Flanagin et al.[34]).

Recommendations for authors or presenters include the following:

1. **Acknowledge the broader social context of health and healthcare disparities when invoking these disparities as a justification for genomic research.** Health disparities are differences in health "closely linked with economic, social, or environmental disadvantage."[60] While health disparities often disproportionately affect minority racial and ethnic groups, the underlying reasons are typically due to social and structural determinants of health rather than genetic factors.[24,61,62] Genetic research may be part of the solution to address health disparities but should be integrated into "social models of disease and interdisciplinary research methods."[25]
2. **Avoid reinforcing the idea that race and ethnicity are genetic concepts when presenting genetically derived data.** When presenting figures or summary statistics, be clear about how labels were defined, use terms that represent the source of the information, and justify their use in the given context. For example, if labeling participants in PC plots by race and/or ethnicity, it is important to state why this was done and use the original racial or ethnic designations rather than re-labeling with (proxy) ancestry terms. As another example, do not assume that allele frequencies from a reference population apply to a particular racial or ethnic group, or vice versa.
3. **Describe participants in alignment with their communities' preferences and study-specific reporting guidelines.** Given the number and complexity of studies with diverse data, and the potential for conflicting study-specific recommendations in cross-study analyses, we encourage authors to discuss these issues with study investigators or participant representatives (e.g., via a community advisory board[63,64]). Where direct access to these stakeholders is infeasible, identify and follow reporting standards or precedents in the study.
4. **Avoid generalizing from a single population to represent another, broader population.** Keep in mind the limitations of population identifiers and generalizability to larger population groups.[65] For example, if a study includes Samoans but no other Pacific Islander populations, do not generalize the Samoan people to represent all Pacific Islanders.

CONCLUSION

Conducting genetic research in the context of large-scale, diverse consortia presents both challenges and opportunities, as illustrated by our experiences in the TOPMed program. Genetics researchers need to make structural changes to the research process and within the scientific community to realize the benefits of diversifying genetics research. We should critically evaluate each research step to ensure that race, ethnicity, and ancestry are described and used appropriately. This includes hypothesis generation, study design, data collection, harmonization, analysis, and reporting. For example, when we set out to identify genetic associations with disease and explore whether differences in association between racial groups exist, it can be easy to conclude that genetic differences rather than social or structural determinants of health are driving observed outcomes. Instead, by incorporating non-genetic factors into an explicit hypothesis up front,[66] we can further address their influence on health disparities.[8] Additionally, measuring and integrating key social and structural factors into genetic analyses

can help elucidate environmental contributions and gene-by-environment interactions.[67–69] It is important to counteract, rather than reinforce, racialized thinking when studying differential health outcomes or group differences.[6]

We recognize our recommendations as part of a broader conversation in the scientific community about refining terminology, strengthening reporting guidelines, and advancing statistical and other research methodologies needed to strive for an anti-racist science.[6,70] Establishing new standards for terminology and incorporating updated publication requirements that demand clear and rigorous definitions of race, ethnicity, and ancestry variables are crucial in extinguishing racialized thinking from genetics research and literature.[8,34,58] These measures encourage investigators to be more critical when applying these concepts in the design, development, and conduct of their research. In addition to changes in language and reporting, methodological advancements that accommodate analyses of diverse populations and a re-evaluation of existing methodologies are necessary.[71] For example, systematic investigation of a stratified versus pooled approach to association testing will provide empirical evidence for if, and when, stratifying participants is necessary. This work is needed because, if used indiscriminately, stratification by race may reify race as genetic and obscure the non-genetic, "fundamental causes" of health inequities.[72]

We should also critically examine the use of continental ancestry in genetic research.[17] The selection of reference populations with ancestry from specific geographic areas is somewhat arbitrary, yet these samples are widely used to represent entire continents.[73] For example, despite early guidance against such oversimplification, the HapMap Yoruba in Ibadan, Nigeria (YRI) are often used to represent all of Africa; however, this population represents a small amount of diversity present across African genomes.[65] Further, the usual classification of people as having European, Asian, American, or African ancestry makes reference to a specific time period, i.e., after the global geographic dispersal of *Homo sapiens* from Africa and prior to the European colonization, especially of the Americas, that accompanied the so-called Age of Discovery. We could just as easily define continental ancestry based on a different time period, such as current human geography.[73] While no more right or wrong, this approach would lead to a very different understanding of, for example, American ancestry. While categorizing ancestry components by continent can be a useful model of the data, we must keep in mind that it is only a model, and one that obscures genetic heterogeneity within continents and the complex, dynamic political, social, and migratory histories of those regions.[74] Scientists are trained to evaluate new data to see if they match expectations, but this training can work against us when it intersects with our social biases because we view results that reflect those biases as more likely to be "true" than other results. This can lead to a belief in the correspondence of continental ancestry with historical races rather than recognizing the practice of clustering genomes in more or fewer population groups as a modeling choice.[75] Allele frequencies and patterns of linkage disequilibrium differ across populations, but these differences are a result of processes including mutation, genetic drift, selective pressure, and population bottlenecks and expansion, reflecting rich population history and migration[73] rather than static genetic differences between a fixed number of population groups.

Averting and correcting misuses of race and ancestry in genetics research now is critical before they potentially get "baked into" emerging applications. One example is the development of polygenic risk scores (PRSs), which provide estimates of an individual's genetic risk for a clinically relevant outcome.[76] PRSs are typically based on summary statistics derived from GWAS data, which to date have been heavily biased toward European populations. This bias has led to poorer predictive performance in non-European and admixed individuals, which could exacerbate health and healthcare disparities.[77] Diversifying study populations in GWASs and developing PRS methods applicable to diverse and admixed populations is of prime importance, but first we need to critically evaluate the roles that race, ethnicity, and ancestry play in these efforts.[78–83] Further, we contend that the recommendations presented here are relevant to PRS development and application, as well as other clinical and translational genomics efforts.[13,15]

Ultimately, awareness, transparency, and sensitivity among researchers are needed to encourage thoughtful data stewardship, foster collaboration, and work toward expanding the diversity and representation needed to further translational genomic research.[1,2,84] As genetic scientists, we should promote meaningful genomic knowledge and scientific advancements with equitable benefit. We should commit to recruiting, supporting, and amplifying the voices of underrepresented scientists in academia and the genetics community more broadly, including internationally.[85] We recognize that addressing race, ethnicity, and ancestry in genetics research is a nuanced practice with changing perspectives. There is much to learn on how best to appropriately consider social factors in genetics research and translation and ensure that we dismantle any remnants of racialized thinking from this work. In order to tackle these issues successfully, we must be open to new and evolving ideas and approach this work with ongoing reflection and humility.

SUPPLEMENTAL INFORMATION

Supplemental information can be found online at https://doi.org/10.1016/j.xgen.2022.100155.

ACKNOWLEDGMENTS

The TOPMed program is supported by the NHLBI. The Data Coordinating Center at the University of Washington has been funded under R01-HL120393, U01-HL120393, and contract HHSN268201800001I. S.T.M. acknowledges grant support from R01-HL093093 and R01-HL133040. A.D.J. acknowledges NHLBI Intramural Funding. B.H. acknowledges grant support from R01-HL155127-01A1. A.T.K., S.M.G., A.M.S., M.L.B., Q.W., S.M.F., M.P.C., and S.C.N. acknowledge grant support from U01HG011697. We are grateful to the GAC Race and Genetics Discussion group and members of the TOPMed ELSI Committee, who provided valuable feedback and input on earlier versions of this work. We also acknowledge our late co-author L. Adrienne Cupples, who passed away in January 2022, for her contributions to this specific work and her decades of leadership in the fields of biostatistics and epidemiology. The views expressed in this manuscript are those of the authors and do not necessarily represent the views of the NHLBI, the National Institutes of Health, or the US Department of Health and Human Services. We gratefully

acknowledge the studies and participants who provided biological samples and data for TOPMed.

AUTHOR CONTRIBUTIONS

Conceptualization, A.T.K., S.M.G., C.P.M., M.P.C., and S.C.N.; writing – original draft, A.T.K., S.M.G., C.P.M., M.P.C., and S.C.N.; writing – review & editing, A.T.K., S.M.G., C.P.M., A.M.S., T.S., M.L.B., Q.W., L.A.C., B.H., A.D.J., M.-L.N.M., S.T.M., M.R.G.T., S.M.F., M.P.C., and S.C.N.

DECLARATION OF INTERESTS

The authors declare no competing interests.

INCLUSION AND DIVERSITY

One or more of the authors of this paper self-identifies as an underrepresented ethnic minority in science. One or more of the authors of this paper self-identifies as a member of the LGBTQ+ community. One or more of the authors of this paper self-identifies as living with a disability.

REFERENCES

1. Popejoy, A.B., and Fullerton, S.M. (2016). Genomics is failing on diversity. Nature *538*, 161–164. https://doi.org/10.1038/538161a.
2. Hindorff, L.A., Bonham, V.L., Brody, L.C., Ginoza, M.E.C., Hutter, C.M., Manolio, T.A., and Green, E.D. (2018). Prioritizing diversity in human genomics research. Nat. Rev. Genet. *19*, 175–185. https://doi.org/10.1038/nrg.2017.89.
3. Bonham, V.L., Warshauer-Baker, E., and Collins, F.S. (2005). Race and ethnicity in the genome era: the complexity of the constructs. Am. Psychol. *60*, 9–15. https://doi.org/10.1037/0003-066x.60.1.9.
4. Race Ethnicity and Genetics Working Group; Genetics Working Group (2005). The use of racial, ethnic, and ancestral categories in human genetics research. Am. J. Hum. Genet. *77*, 519–532. https://doi.org/10.1086/491747.
5. Caulfield, T., Fullerton, S.M., Ali-Khan, S.E., Arbour, L., Burchard, E.G., Cooper, R.S., Hardy, B.-J., Harry, S., Hyde-Lay, R., Kahn, J., et al. (2009). Race and ancestry in biomedical research: exploring the challenges. Genome Med. *1*, 8. https://doi.org/10.1186/gm8.
6. Yudell, M., Roberts, D., DeSalle, R., and Tishkoff, S. (2020). NIH must confront the use of race in science. Science *369*, 1313–1314. https://doi.org/10.1126/science.abd4842.
7. Sirugo, G., Tishkoff, S.A., and Williams, S.M. (2021). The quagmire of race, genetic ancestry, and health disparities. J. Clin. Invest. *131*, 150255. https://doi.org/10.1172/jci150255.
8. Brothers, K.B., Bennett, R.L., and Cho, M.K. (2021). Taking an antiracist posture in scientific publications in human genetics and genomics. Genet. Med. *23*, 1–4.
9. Taliun, D., Harris, D.N., Kessler, M.D., Carlson, J., Szpiech, Z.A., Torres, R., Taliun, S.A.G., Corvelo, A., Gogarten, S.M., Kang, H.M., et al. (2021). Sequencing of 53, 831 diverse genomes from the NHLBI TOPMed Program. Nature *590*, 290–299. https://doi.org/10.1038/s41586-021-03205-y.
10. ASHG denounces attempts to link genetics and racial supremacy. Am. J. Hum. Genet. *103*, 636.
11. Bryc, K., Durand, E., Macpherson, J., Reich, D., and Mountain, J. (2015). The genetic ancestry of African Americans, Latinos, and European Americans across the United States. Am. J. Hum. Genet. *96*, 37–53. https://doi.org/10.1016/j.ajhg.2014.11.010.
12. Nelson, S.C., Yu, J.-H., Wagner, J.K., Harrell, T.M., Royal, C.D., and Bamshad, M.J. (2018). A content analysis of the views of genetics professionals on race, ancestry, and genetics. AJOB Empir. Bioeth. *9*, 222–234. https://doi.org/10.1080/23294515.2018.1544177.
13. Popejoy, A.B., Crooks, K.R., Fullerton, S.M., Hindorff, L.A., Hooker, G.W., Koenig, B.A., Pino, N., Ramos, E.M., Ritter, D.I., Wand, H., et al. (2020). Clinical genetics lacks standard definitions and protocols for the collection and use of diversity measures. Am. J. Hum. Genet. *107*, 72–82. https://doi.org/10.1016/j.ajhg.2020.05.005.
14. Sellers, S.L., Cunningham, B.A., and Bonham, V.L. (2019). Physician knowledge of human genetic variation, beliefs about race and genetics, and use of race in clinical decision-making. J. Racial Ethn. Health Disparities *6*, 110–116. https://doi.org/10.1007/s40615-018-0505-y.
15. Popejoy, A.B., Ritter, D.I., Crooks, K., Currey, E., Fullerton, S.M., Hindorff, L.A., Koenig, B., Ramos, E.M., Sorokin, E.P., Wand, H., et al. (2018). The clinical imperative for inclusivity: race, ethnicity, and ancestry (REA) in genomics. Hum. Mutat. *39*, 1713–1720. https://doi.org/10.1002/humu.23644.
16. Byeon, Y.J.J., Islamaj, R., Yeganova, L., Wilbur, W.J., Lu, Z., Brody, L.C., and Bonham, V.L. (2021). Evolving use of ancestry, ethnicity, and race in genetics research-A survey spanning seven decades. Am. J. Hum. Genet. *108*, 2215–2223. https://doi.org/10.1016/j.ajhg.2021.10.008.
17. Lewis, A.C.F., Molina, S.J., Appelbaum, P.S., Dauda, B., Di Rienzo, A., Fuentes, A., Fullerton, S.M., Garrison, N.A., Ghosh, N., Hammonds, E.M., et al. (2022). Getting genetic ancestry right for science and society. Science *376*, 250–252. https://doi.org/10.1126/science.abm7530.
18. Bonham, V.L., Green, E.D., and Pérez-Stable, E.J. (2018). Examining how race, ethnicity, and ancestry data are used in biomedical research. JAMA *320*, 1533–1534. https://doi.org/10.1001/jama.2018.13609.
19. Alexander, D.H., Novembre, J., and Lange, K. (2009). Fast model-based estimation of ancestry in unrelated individuals. Genome Res. *19*, 1655–1664. https://doi.org/10.1101/gr.094052.109.
20. Price, A.L., Patterson, N.J., Plenge, R.M., Weinblatt, M.E., Shadick, N.A., and Reich, D. (2006). Principal components analysis corrects for stratification in genome-wide association studies. Nat. Genet. *38*, 904–909. https://doi.org/10.1038/ng1847.
21. Roth, W.D., Yaylacı, Ş., Jaffe, K., and Richardson, L. (2020). Do genetic ancestry tests increase racial essentialism? Findings from a randomized controlled trial. PLoS One *15*, e0227399. https://doi.org/10.1371/journal.pone.0227399.
22. Stevens, J. (2003). Racial meanings and scientific methods: changing policies for NIH-sponsored publications reporting human variation. J. Health Polit. Policy Law *28*, 1033–1087. https://doi.org/10.1215/03616878-28-6-1033.
23. Callier, S. (2019). The use of racial categories in precision medicine research. Ethn. Dis. *29*, 651–658. https://doi.org/10.18865/ed.29.s3.651.
24. Williams, D.R., and Sternthal, M. (2010). Understanding racial-ethnic disparities in health: sociological contributions. J. Health Soc. Behav. *51*, S15–S27. https://doi.org/10.1177/0022146510383838.
25. West, K.M., Blacksher, E., and Burke, W. (2017). Genomics, health disparities, and missed opportunities for the Nation's research agenda. JAMA *317*, 1831. https://doi.org/10.1001/jama.2017.3096.
26. Graves, J.L. (2010). Biological V. Social definitions of race: implications for modern biomedical research. Rev. Black Polit. Econ. *37*, 43–60. https://doi.org/10.1007/s12114-009-9053-3.
27. Williams, D.R., and Mohammed, S.A. (2009). Discrimination and racial disparities in health: evidence and needed research. J. Behav. Med. *32*, 20–47. https://doi.org/10.1007/s10865-008-9185-0.
28. Bentley, A.R., Callier, S., and Rotimi, C.N. (2017). Diversity and inclusion in genomic research: why the uneven progress? J. Community Genet. *8*, 255–266. https://doi.org/10.1007/s12687-017-0316-6.
29. Royal, C.D., Novembre, J., Fullerton, S.M., Goldstein, D.B., Long, J.C., Bamshad, M.J., and Clark, A.G. (2010). Inferring genetic ancestry: opportunities, challenges, and implications. Am. J. Hum. Genet. *86*, 661–673. https://doi.org/10.1016/j.ajhg.2010.03.011.
30. Mathieson, I., and Scally, A. (2020). What is ancestry? PLoS Genet. *16*, e1008624. https://doi.org/10.1371/journal.pgen.1008624.

31. Rambachan, A. (2018). Overcoming the racial hierarchy: the history and medical consequences of "Caucasian. J. Racial Ethn. Health Disparities *5*, 907–912. https://doi.org/10.1007/s40615-017-0458-6.

32. Popejoy, A.B. (2021). Too many scientists still say Caucasian. Nature *596*, 463. https://doi.org/10.1038/d41586-021-02288-x.

33. American Psychological Association (2019). APA Style: Racial and Ethnic Identity. https://apastyle.apa.org/style-grammar-guidelines/bias-free-language/racial-ethnic-minorities.

34. Flanagin, A., Frey, T., Christiansen, S.L., and AMA Manual of Style Committee. (2021). Updated guidance on the reporting of race and ethnicity in medical and science journals. JAMA *326*, 621. https://doi.org/10.1001/jama.2021.13304.

35. Stevens, G., Ishizawa, H., and Grbic, D. (2015). Measuring race and ethnicity in the censuses of Australia, Canada, and the United States: parallels and paradoxes. Can. Stud. Popul. *42*, 13. https://doi.org/10.25336/p6pw39.

36. Loveman, M., Muniz, J.O., and Bailey, S.R. (2012). Brazil in black and white? Race categories, the census, and the study of inequality. Ethn. Racial Stud. *35*, 1466–1483. https://doi.org/10.1080/01419870.2011.607503.

37. Long Form Questionnaire (2010 Brazilian Census). https://unstats.un.org/unsd/demographic/sources/census/quest/BRA2010enl.pdf.

38. Gaynor, K., and Williams. (2021). Segregated spaces and separated races: the relationship between state-sanctioned violence, place, and black identity. RSF Russell Sage Found. J. Soc. Sci. *7*, 50.

39. Rothstein, R. (2017). The Color of Law: A Forgotten History of How Our Government Segregated America, First edition (Liveright Publishing Corporation).

40. Ansell, D.A. (2019). The Death Gap: How Inequality Kills.

41. Brown, L.L., Mitchell, U.A., and Ailshire, J.A. (2020). Disentangling the stress process: race/ethnic differences in the exposure and appraisal of chronic stressors among older adults. J. Gerontol. Ser. B *75*, 650–660. https://doi.org/10.1093/geronb/gby072.

42. Krieger, N. (1999). Embodying inequality: a review of concepts, measures, and methods for studying health consequences of discrimination. Int. J. Health Serv. Plan. Adm. Eval. *29*, 295–352. https://doi.org/10.2190/m11w-vwxe-kqm9-g97q.

43. Schisterman, E.F., Cole, S.R., and Platt, R.W. (2009). Overadjustment bias and unnecessary adjustment in epidemiologic studies. Epidemiol. Camb. Mass *20*, 488–495. https://doi.org/10.1097/ede.0b013e3181a819a1.

44. Robinson, L.D., and Jewell, N.P. (1991). Some surprising results about covariate adjustment in logistic regression models. Int. Stat. Rev. *59*, 227. https://doi.org/10.2307/1403444.

45. Sul, J.H., Martin, L.S., and Eskin, E. (2018). Population structure in genetic studies: confounding factors and mixed models. PLoS Genet. *14*, e1007309. https://doi.org/10.1371/journal.pgen.1007309.

46. Wu, C., DeWan, A., Hoh, J., and Wang, Z. (2011). A comparison of association methods correcting for population stratification in case-control studies. Ann. Hum. Genet. *75*, 418–427. https://doi.org/10.1111/j.1469-1809.2010.00639.x.

47. Hellwege, J.N., Keaton, J.M., Giri, A., Gao, X., Velez Edwards, D.R., and Edwards, T.L. (2017). Population stratification in genetic association studies. Curr. Protoc. Hum. Genet. *95*, 1.22.1–1.22.23.

48. Manolio, T.A. (2019). Using the data we have: improving diversity in genomic research. Am. J. Hum. Genet. *105*, 233–236. https://doi.org/10.1016/j.ajhg.2019.07.008.

49. Novembre, J., Johnson, T., Bryc, K., Kutalik, Z., Boyko, A.R., Auton, A., Indap, A., King, K.S., Bergmann, S., Nelson, M.R., et al. (2008). Genes mirror geography within Europe. Nature *456*, 98–101. https://doi.org/10.1038/nature07331.

50. Conomos, M., Laurie, C., Stilp, A., Gogarten, S., McHugh, C., Nelson, S., Sofer, T., Fernández-Rhodes, L., Justice, A., Graff, M., et al. (2016). Genetic diversity and association studies in US hispanic/latino populations: applications in the hispanic community health study/study of latinos. Am. J. Hum. Genet. *98*, 165–184. https://doi.org/10.1016/j.ajhg.2015.12.001.

51. Moreno-Estrada, A., Gignoux, C.R., Fernández-López, J.C., Zakharia, F., Sikora, M., Contreras, A.V., Acuña-Alonzo, V., Sandoval, K., Eng, C., Romero-Hidalgo, S., et al. (2014). Human genetics. The genetics of Mexico recapitulates Native American substructure and affects biomedical traits. Science *344*, 1280–1285. https://doi.org/10.1126/science.1251688.

52. Manichaikul, A., Palmas, W., Rodriguez, C.J., Peralta, C.A., Divers, J., Guo, X., Chen, W.-M., Wong, Q., Williams, K., Kerr, K.F., et al. (2012). Population structure of Hispanics in the United States: the multi-ethnic study of atherosclerosis. PLoS Genet. *8*, e1002640. https://doi.org/10.1371/journal.pgen.1002640.

53. Claw, K.G., Anderson, M.Z., Begay, R.L., Tsosie, K.S., Fox, K., Garrison, N.A., and Summer internship for INdigenous peoples in Genomics (SING) Consortium (2018). A framework for enhancing ethical genomic research with Indigenous communities. Nat. Commun. 9, 2957.

54. Kennedy, B.R., Mathis, C.C., and Woods, A.K. (2007). African Americans and their distrust of the health care system: healthcare for diverse populations. J. Cult. Divers. *14*, 56–60.

55. Streeten, E.A., See, V.Y., Jeng, L.B.J., Maloney, K.A., Lynch, M., Glazer, A.M., Yang, T., Roden, D., Pollin, T.I., Daue, M., et al.; Regeneron Genetics Center (2020). *KCNQ1* and Long QT syndrome in 1/45 amish: the road from identification to implementation of culturally appropriate precision medicine. Circ. Genom. Precis. Med. *13*, e003133. https://doi.org/10.1161/circgen.120.003133.

56. Loos, R.J.F. (2016). CREBRF variant increases obesity risk and protects against diabetes in Samoans. Nat. Genet. *48*, 976–978. https://doi.org/10.1038/ng.3653.

57. Garrison, N.A. (2013). Genomic justice for native Americans: impact of the havasupai case on genetic research. Sci. Technol. Hum. Values *38*, 201–223. https://doi.org/10.1177/0162243912470009.

58. Ali-Khan, S.E., Krakowski, T., Tahir, R., and Daar, A.S. (2011). The use of race, ethnicity and ancestry in human genetic research. HUGO J. *5*, 47–63. https://doi.org/10.1007/s11568-011-9154-5.

59. Fullerton, S.M., Yu, J.-H., Crouch, J., Fryer-Edwards, K., and Burke, W. (2010). Population description and its role in the interpretation of genetic association. Hum. Genet. *127*, 563–572. https://doi.org/10.1007/s00439-010-0800-0.

60. Office of Disease Prevention and Health Promotion Disparities. Disparities. https://www.healthypeople.gov/2020/about/foundation-health-measures/Disparities.

61. Sankar, P. (2004). Genetic research and health disparities. JAMA *291*, 2985. https://doi.org/10.1001/jama.291.24.2985.

62. Meagher, K.M., McGowan, M.L., Settersten, R.A., Fishman, J.R., and Juengst, E.T. (2017). Precisely where are we going? Charting the new terrain of precision prevention. Annu. Rev. Genomics Hum. Genet. *18*, 369–387. https://doi.org/10.1146/annurev-genom-091416-035222.

63. Sorlie, P.D., Avilés-Santa, L.M., Wassertheil-Smoller, S., Kaplan, R.C., Daviglus, M.L., Giachello, A.L., Schneiderman, N., Raij, L., Talavera, G., Allison, M., et al. (2010). Design and implementation of the hispanic community health study/study of latinos. Ann. Epidemiol. *20*, 629–641. https://doi.org/10.1016/j.annepidem.2010.03.015.

64. Lemke, A.A., and Harris-Wai, J.N. (2015). Stakeholder engagement in policy development: challenges and opportunities for human genomics. Genet. Med. *17*, 949–957. https://doi.org/10.1038/gim.2015.8.

65. The International HapMap Consortium (2004). Integrating ethics and science in the international HapMap project. Nat. Rev. Genet. *5*, 467–475. https://doi.org/10.1038/nrg1351.

66. Kaufman, J.S., and Cooper, R.S. (1995). In search of the hypothesis. Public Health Rep. *110*, 662–666.

67. Phuong, J., Riches, N.O., Madlock-Brown, C., Duran, D., Calzoni, L., Espinoza, J.C., Datta, G., Kavuluru, R., Weiskopf, N.G., Ward-Caviness,

CellPress
OPEN ACCESS

C.K., et al. (2022). Social determinants of health factors for gene–environment COVID-19 research: challenges and opportunities. Adv. Genet., 2100056.

68. Mersha, T.B., Qin, K., Beck, A.F., Ding, L., Huang, B., and Kahn, R.S. (2021). Genetic ancestry differences in pediatric asthma readmission are mediated by socioenvironmental factors. J. Allergy Clin. Immunol. *148*, 1210–1218.e4. https://doi.org/10.1016/j.jaci.2021.05.046.

69. Hollister, B.M., Farber-Eger, E., Aldrich, M.C., and Crawford, D.C. (2019). A social determinant of health may modify genetic associations for Blood pressure: evidence from a SNP by education interaction in an african American population. Front. Genet. *10*, 428. https://doi.org/10.3389/fgene.2019.00428.

70. National Academies of Sciences, Engineering, Medicine (2022). Use of Race, Ethnicity, and Ancestry as Population Descriptors in Genomics Research. https://www.nationalacademies.org/our-work/use-of-race-ethnicity-and-ancestry-as-population-descriptors-in-genomics-research.

71. Peterson, R.E., Kuchenbaecker, K., Walters, R.K., Chen, C.-Y., Popejoy, A.B., Periyasamy, S., Lam, M., Iyegbe, C., Strawbridge, R.J., Brick, L., et al. (2019). Genome-wide association studies in ancestrally diverse populations: opportunities, methods, pitfalls, and recommendations. Cell *179*, 589–603. https://doi.org/10.1016/j.cell.2019.08.051.

72. Phelan, J.C., and Link, B.G. (2005). Controlling disease and creating disparities: a fundamental cause perspective. J. Gerontol. B Psychol. Sci. Soc. Sci. *60*, 27–33. https://doi.org/10.1093/geronb/60.special_issue_2.s27.

73. Biddanda, A., Rice, D.P., and Novembre, J. (2020). A variant-centric perspective on geographic patterns of human allele frequency variation. Elife *9*, e60107. https://doi.org/10.7554/elife.60107.

74. Belbin, G.M., Cullina, S., Wenric, S., Soper, E.R., Glicksberg, B.S., Torre, D., Moscati, A., Wojcik, G.L., Shemirani, R., Beckmann, N.D., et al. (2021). Toward a fine-scale population health monitoring system. Cell *184*, 2068–2083.e11. https://doi.org/10.1016/j.cell.2021.03.034.

75. Roberts, D.E. (2011). Chapter 3: redefining race in genetic terms. In Fatal invention: how science, politics, and big business re-create race in the twenty-first century (New Press).

76. Wand, H., Lambert, S.A., Tamburro, C., Iacocca, M.A., O'Sullivan, J.W., Sillari, C., Kullo, I.J., Rowley, R., Dron, J.S., Brockman, D., et al. (2021). Improving reporting standards for polygenic scores in risk prediction studies. Nature *591*, 211–219. https://doi.org/10.1038/s41586-021-03243-6.

77. Martin, A.R., Kanai, M., Kamatani, Y., Okada, Y., Neale, B.M., and Daly, M.J. (2019). Current clinical use of polygenic scores will risk exacerbating health disparities. Nat. Genet. *51*, 584–591. https://doi.org/10.1038/s41588-019-0379-x.

78. Amariuta, T., Ishigaki, K., Sugishita, H., Ohta, T., Koido, M., Dey, K.K., Matsuda, K., Murakami, Y., Price, A.L., Kawakami, E., et al. (2020). Improving the trans-ancestry portability of polygenic risk scores by prioritizing variants in predicted cell-type-specific regulatory elements. Nat. Genet. *52*, 1346–1354. https://doi.org/10.1038/s41588-020-00740-8.

79. Atkinson, E.G., Maihofer, A.X., Kanai, M., Martin, A.R., Karczewski, K.J., Santoro, M.L., Ulirsch, J.C., Kamatani, Y., Okada, Y., Finucane, H.K., et al. (2021). Tractor uses local ancestry to enable inclusion of admixed individuals into GWAS and boost power. Nat. Genet. *53*, 195–204. https://doi.org/10.1038/s41588-020-00766-y.

80. Cai, M., Xiao, J., Zhang, S., Wan, X., Zhao, H., Chen, G., and Yang, C. (2021). A unified framework for cross-population trait prediction by leveraging the genetic correlation of polygenic traits. Am. J. Hum. Genet. *108*, 632–655. https://doi.org/10.1016/j.ajhg.2021.03.002.

81. Weissbrod, O., Kanai, M., Shi, H., Gazal, S., Peyrot, W.J., Khera, A.V., Okada, Y., Project, T.B.J., Martin, A.R., Finucane, H., et al. (2021). Leveraging fine-mapping and non-European training data to improve cross-population polygenic risk scores. Preprint at medRxiv. https://doi.org/10.1101/2021.01.19.21249483.

82. Ruan, Y., Lin, Y.-F., Feng, Y.-C.A., Chen, C.-Y., Lam, M., Guo, Z., Initiatives, S.G.A., He, L., Sawa, A., Martin, A.R., et al. (2021). Improving polygenic prediction in ancestrally diverse populations. Nat. Genet. *54*, 573–580.

83. NIH Polygenic Risk Methods in Diverse Populations (PRIMED) Consortium. https://primedconsortium.org/.

84. Sirugo, G., Williams, S.M., and Tishkoff, S.A. (2019). The missing diversity in human genetic studies. Cell *177*, 1080. https://doi.org/10.1016/j.cell.2019.04.032.

85. Cooper, R.S., and Rotimi, C.N. (2020). The practice of anti-racist science requires an internationalist perspective. Am. J. Hum. Genet. *107*, 793–796. https://doi.org/10.1016/j.ajhg.2020.09.008.

Cell Genomics, Volume *2*

Supplemental information

Recommendations on the use and reporting of race, ethnicity, and ancestry in genetic research: Experiences from the NHLBI TOPMed program

Alyna T. Khan, Stephanie M. Gogarten, Caitlin P. McHugh, Adrienne M. Stilp, Tamar Sofer, Michael L. Bowers, Quenna Wong, L. Adrienne Cupples, Bertha Hidalgo, Andrew D. Johnson, Merry-Lynn N. McDonald, Stephen T. McGarvey, Matthew R.G. Taylor, Stephanie M. Fullerton, Matthew P. Conomos, and Sarah C. Nelson

Additional background

From 2014-2020, the TOPMed Data Coordinating Center (DCC) was housed in the Genetic Analysis Center (GAC) in the Department of Biostatistics at the University of Washington[1]. The GAC has performed scientific, analytical, and/or administrative coordination for a range of human genomics consortia and programs over the past 15 years including the NHGRI Gene Environment Association Studies consortium (GENEVA, 2007-2011), NHGRI Genomics and Randomized Trials Network (GARNET, 2009-2012), and the NHLBI Hispanic Community Health Study/Study of Latinos (HCHS/SOL, 2013-2016). Through these efforts, we have established standards for genotypic data quality assurance that account for population structure[2], grappled with how to analyze and report genetic diversity, e.g. among Hispanic/Latino groups[3], and developed statistical methods and software for analyzing diverse datasets[4–7]. In 2018, GAC staff initiated monthly internal discussions on the use of race, ethnicity, and ancestry in genetics research—engaging with academic literature, public media, and our own experiences working in TOPMed and prior genetics consortia. We discussed a variety of articles across disciplines[8–11] and invited guest speakers on topics such as statistical rationale for stratified analyses and the co-opting of population genetic research by white supremacists on social media[12]. Discussions were informed by a range of training and experience at the DCC, including biostatistics; statistical genetics; science communication; public health genetics; and ethical, legal, and social implications (ELSI). From these discussions, we recognized the opportunity as a DCC to help establish recommendations for TOPMed researchers that address the challenges of working with diverse data and incorporate anti-racist principles[13] into the research process.

References

1. Genetic Analysis Center https://www.biostat.washington.edu/research/centers/gac.
2. Laurie, C.C., Doheny, K.F., Mirel, D.B., Pugh, E.W., Bierut, L.J., Bhangale, T., Boehm, F., Caporaso, N.E., Cornelis, M.C., Edenberg, H.J., et al. (2010). Quality control and quality assurance in genotypic data for genome-wide association studies. Genet. Epidemiol. *34*, 591–602.
3. Conomos, M.P., Laurie, C.A., Stilp, A.M., Gogarten, S.M., McHugh, C.P., Nelson, S.C., Sofer, T., Fernández-Rhodes, L., Justice, A.E., Graff, M., et al. (2016). Genetic Diversity and Association Studies in US Hispanic/Latino Populations: Applications in the Hispanic Community Health Study/Study of Latinos. Am. J. Hum. Genet. *98*, 165–184.
4. Conomos, M.P., Miller, M.B., and Thornton, T.A. (2015). Robust inference of population structure for ancestry prediction and correction of stratification in the presence of relatedness. Genet. Epidemiol. *39*, 276–293.
5. Conomos, M.P., Reiner, A.P., Weir, B.S., and Thornton, T.A. (2016). Model-free Estimation of Recent Genetic Relatedness. Am. J. Hum. Genet. *98*, 127–148.
6. Gogarten, S.M., Sofer, T., Chen, H., Yu, C., Brody, J.A., Thornton, T.A., Rice, K.M., and Conomos, M.P. (2019). Genetic association testing using the GENESIS R/Bioconductor package. Bioinforma. Oxf. Engl. *35*, 5346–5348.
7. Chen, H., Wang, C., Conomos, M.P., Stilp, A.M., Li, Z., Sofer, T., Szpiro, A.A., Chen, W., Brehm, J.M., Celedón, J.C., et al. (2016). Control for Population Structure and Relatedness

for Binary Traits in Genetic Association Studies via Logistic Mixed Models. Am. J. Hum. Genet. *98*, 653–666.

8. Reich, D. (2018). How Genetics Is Changing Our Understanding of 'Race.' N. Y. Times.
9. Roth, W.D., and Ivemark, B. (2018). Genetic Options: The Impact of Genetic Ancestry Testing on Consumers' Racial and Ethnic Identities. Am. J. Sociol. *124*, 150–184.
10. Royal, C.D., Novembre, J., Fullerton, S.M., Goldstein, D.B., Long, J.C., Bamshad, M.J., and Clark, A.G. (2010). Inferring Genetic Ancestry: Opportunities, Challenges, and Implications. Am. J. Hum. Genet. *86*, 661–673.
11. Mills, M.C., and Rahal, C. (2019). A scientometric review of genome-wide association studies. Commun. Biol. *2*, 9.
12. Carlson, J., and Harris, K. (2020). Quantifying and contextualizing the impact of bioRxiv preprints through automated social media audience segmentation. PLOS Biol. *18*, e3000860.
13. Brothers, K.B., Bennett, R.L., and Cho, M.K. (2021). Taking an antiracist posture in scientific publications in human genetics and genomics. Genet. Med.
